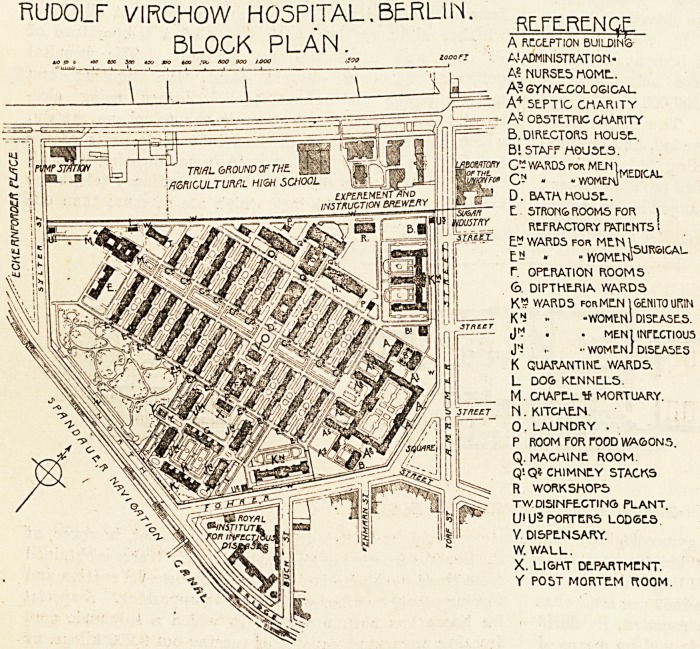# Some Modern Continental Hospitals

**Published:** 1908-08-01

**Authors:** 


### HOSPITAL ADMINISTRATION.

### CONSTRUCTION AND ECONOMICS.

### SOME MODERN CONTINENTAL HOSPITALS.

#### I.—THE RUDOLPH VIRCHOW HOSPITAL AT BERLIN.

In 1898 it was decided to build a fourth communal hospital of Berlin as a tribute to the memory of the great German pathologist, Virchow. Plans were prepared by Geheim Baurat Dr. Hoffmann, and in the spring of 1899 actual building operations were started on a site in the northern quarter of the city. The first part of the building was completed in October, 1906, but even at present the whole building is not quite finished, and the details of the total cost of construction are consequently merely approximate. Those who are interested in bare statistics may find the following figures, culled from the last "Verwaltungsbericht" of the Town Council, of some interest. "Area covered by hospital and surrounded by wall W (see ground plan), 25.7 hectares; number of beds available for in-patients, 2,000; number of separate buildings, 57; approximate cost of construction, 19,100,000 marks; approximate cost per bed, 9,550 marks." This construction cost, it may be mentioned, has been exceeded, and the total outlay is nearer a million pounds sterling.

The hospital has been built on a modified pavilion system, a feature being that the wards are all arranged in one-storied pavilions, only the dermatological, gynæcological, and obstetric departments being located in buildings of more than one story in height. The main buildings have a quiet, dignified appearance, and the whole reminds one somewhat of the Louvre palace, with its corners rounded, its florid ornamentation subdued, and its walls cleaned by means of suction machines.

The administration block is large and well arranged, comprising, on the ground floor, a series of large working and waiting rooms, rooms for the administrative staff, and extra and smaller rooms. A large and very fine staircase, wide and with low steps of granite, with ornamental wrought-iron work balusters, leads to the first and second floors, which are connected by corridors with the gynæcological pavilions. To the left of the administrative buildings, but in the same block, are the rooms for the nursing staff. On the second floor is a large and very fine reception hall for meetings and special demonstrations. On the first floor are the rooms for the staff, the working rooms (studies and special sitting rooms) being labelled with the name and rank of the officials. Throughout this block the rooms have been excellently finished; the walls are of smooth cement, painted with special distemper. The fittings are of brass or painted steel. The heating is by steam pipes, there being a large radiator in each room. The windows are double, and, like the doors, are made more or less dust proof. The electric and boiler department is housed in a special building some distance to the right. There are 11 low and 5 high pressure boilers of large capacity, which are used both for heating and for power purposes. In the wards the heating is throughout by hot water pipes; in other parts by steam pipes. The ventilation is to a large extent mechanical, several large ventilating fans being employed, but in the wards natural ventilation is used as well. There is a large and very interesting hot water apparatus in the machine room. This apparatus provides the whole establishment, from administrative block to autopsy room, at all times, whether night or day, with water of a temperature of 70° C., and at a uniform pressure. The hospital possesses its own electric plant, the lighting and motive power throughout the buildings being electrical. An interesting feature is the telephone system, which has 100 different stations in various parts of the building, so that every part is in communication with all the others, and also directly with the ordinary telephonic system. In those buildings which are of more than one story in height electric lifts are provided, not, however, of the fine pattern used in London hospitals. Water is obtained from three deep artesian wells, and a powerful suction and forcing plant, together with filtration apparatus. A special ice house has been provided, in which a sulphuric acid freezing apparatus, capable of turning out 2,200 kilogs. of ice per day, is working efficiently.

RUDOLF VIRCHOW HOSPITAL. BERLIN.

GROUND PLAN OF ONE WARD BLOCK.

The drainage system has been very carefully seen to, and is very efficient. The drains all communicate with the main city drains, but a special disinfecting drain has been interposed between the main drain pipe and the pipes issuing from the isolation block and the post-mortem room. The disinfection rooms are placed at some distance from the laundry house, but each ward has a small disinfecting room attached to it. The laundry house is a very large and airy-roomed building. Equally efficient is the kitchen, which is unusually well lit, and furnished with a wonderful series of labour-saving machines. These are all driven by electricity, and include sausage-stuffing, potato-peeling (five), mincing, meat-carving, and mashing machines. There are 27 boilers, besides steaming machines, ovens, grids, and an attached bakery. The food is put in specially-constructed water-jacketed cans, which are moved to the various pavilions by specially-designed "food carts," light, zinc-lined arrangements on wheels, which are easily drawn by a single man.

These carts are kept in a specially heated room attached to the main kitchen. Each ward, in addition, has its own kitchen.

All the buildings are connected subterraneally by means of a series of galleries and gangways, rendering it possible in bad weather to move patients easily from any part of the institution to any other part. The floors in the working rooms are of cement or of marble tiles, and are cleaned by means of flushing and mopping. The pavilions are very large, and perhaps too close to each other, 20 metres of lawn separating each from the other. The central portion is double storied, the upper story containing the sleeping and sitting-rooms of the staff attached to the pavilion. On the ground floor is the entrance hall, with a rather narrow doorway, which does not permit of ideal ventilation. One feels cramped in the central hall, with its three feet four doorway, after going through the lofty rooms of the administrative block. On each side are the special rooms : a kitchen, a scullery, a laboratory, a small disinfecting room for the patients' laundry and clothing, a large day room, a dressing room, and examination room : the night nurse's sitting room, and an apparatus room. All these are fairly large, clean, and comfortable, with plenty of locker accommodation. The walls are cement and the floors tiled. Throughout the institution scrupulous cleanliness appears to be the rule, and the hose and the suction cleaning apparatus are employed daily. The fault of this central part is that it contains too many rooms, the general impression that one gets being that there has been too great economy of space.

RUDOLF VIRCHOW HOSPITAL. BERLIN.

BLOCK PLAN.

REFERENCE

A RECEPTION BUILDING.

$A^1$ ADMINISTRATION.

$A^2$ NURSES HOME.

$A^3$ GYNAECOLOGICAL

$A^4$ SEPTIC CHARITY

$A^5$ OBSTETRIC CHARITY

B. DIRECTORS HOUSE.

$B^1$ STAFF HOUSES.

$C^m$ WARDS FOR MEN } MEDICAL

$C^n$ " " WOMEN }

D. BATH HOUSE.

E. STRONG ROOMS FOR REFRACTORY PATIENTS

$E^m$ WARDS FOR MEN } SURGICAL

$E^n$ " " WOMEN }

F. OPERATION ROOMS

G. DIPTHERIA WARDS

$K^m$ WARDS FOR MEN } GENITO URIN

$K^n$ " " WOMEN } DISEASES.

$J^m$ " " MEN } INFECTIOUS

$J^n$ " " WOMEN } DISEASES

K QUARANTINE WARDS.

L DOG KENNELS.

M. CHAPEL & MORTUARY.

N. KITCHEN.

O. LAUNDRY.

P ROOM FOR FOOD WAGONS.

Q. MACHINE ROOM.

$Q^1$ $Q^2$ CHIMNEY STACKS

R WORKSHOPS

TW. DISINFECTING PLANT.

$U^1$ $U^2$ PORTERS LODGES.

V. DISPENSARY.

W. WALL.

X. LIGHT DEPARTMENT.

Y POST MORTEM ROOM.

A small corridor leads directly into the ward on each side of the central hall. Each ward is cheerful and comfortable looking, the walls and ceiling—the latter a trifle low, perhaps—are painted blue-green below, and white above, relieved by floral designs in red and yellow. The heating is by steam radiators, and the beds, twenty in each ward, are ranged between each window, fairly close to the wall. The cosy appearance of the ward is heightened by the liberal display of flowers and foliage plants ranged on iron stands along the broad central way. The ward utensils and instruments are kept at the end, close to the sister's room, which adjoins the ward. The windows, even on the surgical side, have yellow dimity curtains, which are certainly not without dust. The windows themselves are rather high from the floor, but it is stated that they have been carefully tried, and that they are quite efficient, on a sunny day, to flood every square inch of the ward floor with direct sunlight. The visitor acquainted with English hospitals is at once struck with the fact that all extras in the ward have been reduced to the minimum. Except for the beds, the bed tables, the flower stands, the few instrument and medicine shelves, there is practically no ward furniture—no lockers, no bookcases, and no window sills littered with odds and ends. The patients, on entering the ward, are undressed and washed in the reception room. Their clothing is at once taken to the small disinfecting room, disinfected as a matter of routine, and transferred to the large underground "clothing press" in the laundry department, where it remains until the patients leave the hospital. While an inmate the patient wears hospital clothing—flannel underclothing, and a woollen dress of a sober but not unhandsome pattern. The total cost of such an outfit is about 18s., and patients do not object to it, as they did at the Bradford Infirmary.

From the wards a long corridor leads to the emergency exit at the other end of the pavilion. On this corridor open the doors of the various rooms placed at each end. They are small, single-bedded, separate, isolation rooms (each pavilion accommodates 47 patients in all), a large bathroom fitted with hot and cold baths, and a spray and douche bath as well, a lavatory, and at the end two water closets. It is this corridor that may give rise to most criticism. Its length and comparative darkness are to be regretted, and it in fact makes the pavilion into a hospital of the corridor type, with its attendant disadvantages. Another defect is that to reach the closets the patients have to traverse the whole length of this corridor, and to pass the isolation rooms. The latter, again, are separated by too wide an interval from the sister's and nurses' rooms, though they are, of course, in communication with these by means of bells and telephones. The long end corridor receives no light from any window—only from the doors of the rooms that open on to it. These objections apart, the arrangements in this end block are admirable. The drainage is very effective, and no smell is to be perceived. The hand basins and baths are scrupulously clean, and the douche, when we tried it, worked to perfection. One missed the tip basins which are perhaps ideal, allowing a free flushing into the drain, and a feature possibly open to objection is that the basin pipes are very narrow and covered over by a fixed and perforated grating, which must make their effective cleaning somewhat difficult.

The wards in the other departments do not differ materially, though in the gynæcological block they are placed below a series of rooms used for various purposes. In every ward the patients looked neat and clean. Screens, which are of simple canvas stretched on steel frames, are used only when necessary, and are not kept in the wards.

(*To be continued.*)

## Figures and Tables

**Figure f1:**
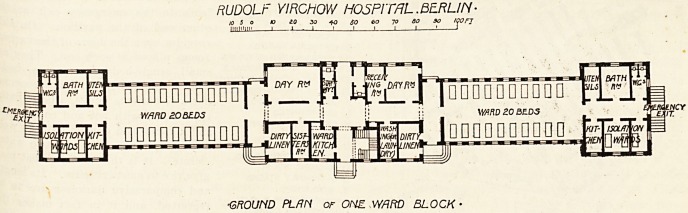


**Figure f2:**